# Supplementary material for: Replacing quarantine of COVID-19 contacts with periodic testing is also effective in mitigating the risk of transmission
Source: Sci Rep. 2022 Mar 7;12:3620. doi: 10.1038/s41598-022-07447-2 (PMC8901648; doi:10.1038/s41598-022-07447-2)
Supplement: Supplementary file 1 — Supplementary Information. [file 41598_2022_7447_MOESM1_ESM.pdf]

Appendix: Mathematical Formulation

A Disease Evolution and Infectiousness

Since the moment a susceptible person becomes infected with COVID-19, the viral load steadily increases until it reaches the limit of detection (LOD), which is the minimum viral load that can be detected with a PCR test. Then, the viral load keeps growing until reaching its peak value. After that, it continuously decreases until it becomes undetectable. Following [2], we use a LOD of 10^3 cp/ml for PCR tests and 10^5 cp/ml for LFA tests. This methodology adjusts the parameters to simulate viral load for symptomatic and asymptomatic patients.

For the viral load evolution, we use the model described in [2], and use V_t to denote the viral load of an infected individual at time t since exposure to the virus. The following parameters describe the control points used to generate sample paths of V_t (see Figure 1):

- t_0= Time when an infected person reaches the LOD of 10^3 cp/ml of viral load. $t_0 \sim U[2.5, 3.5]$.
- t_{peak} = Time when an infected individual reaches peak viral load. $t_{peak} = t_0 + \min(3, (0.5 + \gamma))$, where γ is a random variable that follows a gamma distribution with a shape of 1.5 and scale of 1.
- V_{peak} = Value of the logarithm of viral load at its peak. $V_{peak} = V_{t_{peak}} \sim U[7, 11]$.
- t_{sympt}= Time when symptoms appear for a symptomatic individual. An individual is symptomatic with probability 1/2. $t_{sympt} = t_{peak} + s$, where $s \sim U[0, 3]$.
- t_f: Ending time of infectious period. The probability distribution of t_f depends on whether the individual is symptomatic or not. Thus, if it is symptomatic, then $t_f = t_{sympt} + f$; if it is asymptomatic, then $t_f = t_{peak} + f$, where $f \sim U[4, 9]$. Note that this is the time when the viral load drops below 10^6 and the individual is no longer infectious.

We assume that the logarithm of the viral load follows a linear function (constant growth) between the time it reaches the LOD for PCR and the time of peak load (from 3 to V_{peak})) and another linear function (constant decrease) from the peak time to the end time of infection, from V_{peak} to 6. These model parameters fully characterize the viral load evolution for both a symptomatic and an asymptomatic patient in the course of the disease.

B Mathematical Formulation

Our goal is to determine the most effective testing policies for traced contact to minimize the secondary infection risk in the community, without the need of an immediate isolation. Thus, we minimized the expected number of contagious days (i.e. days in which the contact is infecting, with viral load greater than or equal to 10^6 cp/ml), and the traced contact was active in the community before detection and isolation.

Let T be the time horizon in days in which we monitor the traced contact (set to $T = 14$). Thus, a test can be scheduled on any day $t = 0, 1, \ldots, T$, where $t = 0$ corresponds to the day on which the contact is identified. A testing schedule is defined by the specific days in which the LFA and/or PCR tests are taken: we denote a schedule as a tuple (P, A), where A is the set of days an LFA test is performed, and P is the set of days a PCR test is performed. If we fix the number of LFA and PCR tests that can be used, then the set $\Pi(i, j)$ consists of all feasible schedules to perform exactly j LFA tests and i PCR tests. We notice that all policies within this set use exactly the same number of tests of each type. For each schedule, we compute the expected number of infectious days a traced contact is active in the community before being isolated. Additionally, we denote $\Pi = \cup_{i,j \in \mathbb{N}} \Pi(i, j)$ as the set of all possible policies.

The dynamics of the testing and isolation process are as follows. At the beginning of t, an LFA or PCR test is taken if they are scheduled at t. In the case of a LFA test, we assume that its result is observed immediately. In practice, this would take at most 30 minutes, but we assume that the individual is isolated until the LFA test result is back, which makes this is a realistic assumption; in the case of a PCR test, we observe the test result at the beginning of the next day ($t + 1$). When positive test results are observed, the individual is immediately isolated and starts quarantine. If the test result is negative, then the individual remains active in the community until the next scheduled test or if symptoms develop.

We evaluated the performance of a test schedule based on the number of days a suspected infected individual was contagious before being identified as such and therefore imposed a risk to the community. For this, we take the perspective of a decision maker who has a budget that specifies the number of LFA and PCR tests that can be performed and needs to decide on which days to take these tests to minimize the number of days an infected individual was contagious in the workplace before being isolated. We remark on the difference between being infected and being contagious, and only the latter imposes an exposure risk to others.

There are several sources of randomness when measuring the number of infectious days of a traced contact before isolation. In what follows, we explain how we consider this randomness in our model and its effect on the computation of the expected number of infectious days.

- **Uncertainty on whether or not a traced contact has been infected:** in our analysis, we assume the individual *is* actually infected (i.e., we condition on the event that the contact was infected by the index case at some date of exposure). Although this may seem paradoxical at first since an infected individual should always be isolated, this is methodologically correct in our setting. Our optimization minimizes the number of infecting days subject to the individual not being isolated until confirmed by a positive test or self-isolated at symptom onset. In this optimization, a non infected individual will always contribute zero to the infecting days regardless of the selected policy; therefore, the expected value is conditional on the event of infection. Another reason is that given the applications we consider, we believe decision makers are more concerned with measuring the performance of a testing policy with respect to how good it is at isolating infecting individuals rather than focusing on cases in which the individual is actually not infected. Additionally, taking this approach makes us indifferent to the underlying probability of being infected, which is difficult to estimate and context dependent.

  Formally, the objective is to minimize is the expected number of infecting days, and our control is the schedule of the tests (given a number of tests). Denote π the testing policy (days in which the tests are performed), $N_{inf}(\pi)$ a random variable representing the number of infecting days under that policy and $\mathbb{E}[N_{inf}(\pi)]$ its expectation. Because $N_{inf}(\pi)$ equals to zero when the contact was not infected, conditioning on the event that the contact is infected yields:

  $$\mathbb{E}[N_{inf}(\pi)] = \mathbb{E}[N_{inf}(\pi) \mid \text{Contact } \textbf{is} \text{ infected}] \times \Pr(\text{Contact } \textbf{is} \text{ infected})$$

  Because the probability that the contact is infected ($\Pr(\text{Contact } \textbf{is} \text{ infected})$) is independent of the testing policy, choosing π to minimize the expected number of infecting days is equivalent to minimize the conditional expectation $\mathbb{E}[N_{inf}(\pi) \mid \text{Contact } \textbf{is} \text{ infected}]$. Hence, the optimal testing policy is independent on the prior probability that the contact is infected. We focus the optimization to minimize the number of infected days given that the individual is infected; scaling this objective by the prior probability of infection yields the (unconditional) expected number of infecting days.

- **Number of infecting days of the infected contact:** The generative model for the viral load was described in Appendix A and modeled as a function of time, generating multiple curves randomly based on the controls points described in Figure 1 . The expected number of infecting days (N_{inf}) is calculated for each simulated viral load path, considering the period before isolation (either through a positive test or self-isolation of symptomatic cases at the onset of symptoms). Hence, this methodology is flexible to accommodate alternative approaches to generate the viral load curve.

- **Day of infection of the contact:** Our methodology incorporates uncertainty on the day in which the contact has been infected, assuming a set of days where the index case and the contact had significant interaction and the day the index case was confirmed as infected. This assumption is more realistic in settings with structured contact networks that interact frequently (e.g. school and workplace).

  To incorporate this uncertainty in the model, we build a probabilistic distribution of the days in which the infection may have happened, and use this probability distribution when simulating the viral loads of the contacts. Transmission from the index case to the contact occurs on exposure day t when: (i) the index case is infectious on day t (defined as the event I_t); (ii) the contact has not yet been infected by the index, that is, is susceptible at the beginning of day t (defined as the event S_t). Conditional on the events I_t and S_t, infection occurs with probability β, referred to as the infectivity parameter. Using these definitions, the probability that the index case transmits the disease to the contact on day t is given by:

$$r_t = \beta \Pr(I_t|S_t) \cdot \Pr(S_t), \tag{1}$$

  Note that the events I_t and S_t are not independent, because observing no infection prior to t provides some evidence that the index case may have not yet been infectious. Hence, we use simulation methods to compute equation (1).

  Define the event U_d = index case was infected on day d. For each day d prior to the index case confirmation, we simulate many viral load paths representing the evolution of the disease for the index case when he/she was infected on day d. Each viral load path $V^{k,d}$ is a vector specifying the viral load of the index case on each day, denoted $V_t^{k,d}$ (set to zero for $t < d$ because the index was not yet infected); with some abuse of notation, we also use $V^{k,d}$ to denote the event that the index case follows this viral load path. A priori, all the paths $V^{k,d}$ have the same probability, but conditioning on index case confirmation at $t = 0$ generates a filter that removes paths that are not consistent with the confirmation event. For example, when the index case is detected via a random LFA test, the filter drops all the paths with $V_0^{k,d} < 10^5$; for the weekly LFA test detection, an additional filter is used to drop all the paths with $V_{-6}^{k,d} > 10^5$. Denote $\tilde{V}^{k,d}$ all the paths that *remain* after the filters, $\tilde{N}$ the total number of remaining paths and u_d the number of these paths that start on day d. Note that confirmation at $t = 0$ implies that in all the surviving paths the contact has not yet self-isolated during $t < 0$. The conditional probability that the index case was infected on day d, is the proportion of paths $\tilde{V}^{k,d}$ that start on day d, defined as $q_d = u_d/\tilde{N}$.

  Conditioning on the filtered paths $\tilde{V}^{k,d}$ and using the indicator function $\mathbb{1}(\tilde{V}_t^{k,d} > 10^6)$ to represent a viral path that is infectious on day t, equation (1) can be expressed as:

$$\begin{aligned} r_t &= \frac{1}{\tilde{N}} \sum_{k,d} \beta \Pr(I_t|S_t, \tilde{V}^{k,d}) \cdot \Pr(S_t|\tilde{V}^{k,d}) \\ &= \frac{1}{\tilde{N}} \sum_{k,d} \beta \mathbb{1}(\tilde{V}_t^{k,d} > 10^6) \cdot \prod_{j \le t-1} (1 - \beta \mathbb{1}(\tilde{V}_j^{k,d} > 10^6)) \\ &= \sum_{d \le t} q_d \cdot \frac{1}{u_d} \sum_{k} \beta \mathbb{1}(\tilde{V}_t^{k,d} > 10^6) \cdot \prod_{j \le t-1} (1 - \beta \mathbb{1}(\tilde{V}_j^{k,d} > 10^6)) \end{aligned} \tag{2}$$

To facilitate computations, we used the following approximation for equation (2):

$$r_t \approx \sum_{d \le t} q_d \cdot \beta \frac{1}{u_d} \sum_k \mathbb{1}(\tilde{V}_t^{k,d} > 10^6) \cdot \prod_{j \le t-1} (1 - \beta \frac{1}{u_d} \sum_k \mathbb{1}(\tilde{V}_j^{k,d} > 10^6))$$
$$= \sum_d q_d \cdot \beta \Pr(I_t|U_d) \cdot \prod_{j \le t-1} (1 - \beta \Pr(I_j|U_d)),$$

where the values $\Pr(I_j|U_d) = \frac{1}{u_d} \sum_k \mathbb{1}(\tilde{V}_t^{k,d} > 10^6)$ can be computed once and used for all the simulations including different values of the infectivity parameter β. Finally, we compute the normalized probabilities by conditioning that the contact was infected. For the results shown in this paper, we have computed the exact and approximate values of the normalized r_t for all t and high and low values of β, and obtained good approximations, within 1% of the probability values.

C Simulation based optimization

In what follows, we present a detailed mathematical formulation for the optimization problem. We consider a standard probability space in which we measure the viral load of an individual who has been infected. This randomness could be attributed to the random variations in viral load evolution for different individuals. All random variables and filters are defined with respect to this probability space. We use the following notation:

- V_t, $t = 1, \ldots, T$ = Viral load on day t after the index case is discovered. If we know the exact day the individual was infected, then V_t would be completely described by the process explained in Section A. However, since we do not necessarily know the exact day but only a probabilistic distribution over the days of infection, we take V_t to be the random process conditioned on that infection day distribution.
- x_t^A, x_t^P = Variables indicating that a test result was **observed** at day t (x^A for LFA test, and x^P for PCR), and they have a value of 1 if a result is observed (independent of its value) and 0 otherwise.
- $L^A = 10^5$, $L^P = 10^3$ Levels of detection for each test type. We use and $L^I = 10^6$ to represent the viral load threshold above which an individual is infectious.
- $\mathcal{D}^A = \{d \mid x_d^A = 1\}$, $\mathcal{D}^P = \{d \mid x_d^P = 1\}$. Sets of days where a LFA and PCR test result were observed. Note that for the LFA test, this value coincides with the day of the test, whereas for PCR, it corresponds to one day later (we assume that PCR test results are obtained 24 hours after they are taken, while for LFA tests, these are obtained immediately). Thus,
- $\mathcal{D}_t^A\{d \in \mathcal{D}^A \mid d \le t\}$: Days of LFA test results up to day t. Similar for $\mathcal{D}_t^P$ with PCR test days.
- R_t^A, R_t^P = Random variables indicating the result of an LFA or PCR test observed on day t. The distributions of R_t^A and R_t^P depend on x_t^A and x_t^P, respectively. If a test result was not observed that day, then we assume that R_t takes the value of -1.

$$R_t^A = \begin{cases} -1 & x_t^A = 0 \\ \mathbb{1}\{V_t \ge L^A\} & x_t^A = 1 \end{cases}, \quad R_t^P = \begin{cases} -1 & x_t^P = 0 \\ \mathbb{1}\{V_{t-1} \ge L^P\} & x_t^P = 1 \end{cases},$$

Denoting $R_t = \max(R_t^A, R_t^P)$ as a random variable that indicates the presence of any test, then:

$$R_t = \begin{cases} -1 & x_t^A = x_t^P = 0 \\ \mathbb{1}\{V_t \ge L^A\} & x_t^A = 1 \text{ and } x_t^P = 0 \\ \mathbb{1}\{V_{t-1} \ge L^P\} & x_t^A = 0 \text{ and } x_t^P = 1 \\ \mathbb{1}\{V_t \ge L^A \text{ or } V_{t-1} \ge L^P\} & x_t^A = x_t^P = 1. \end{cases}$$

- $\mathcal{H} = (\mathcal{H}_t)_t$ = Filtration with respect to the process $((R_t^A, R_t^P))_t$, i.e., $\mathcal{H}_t = \sigma(R_k^A, R_k^P \mid k \leq t)$.

- $\mathcal{S}_t$ = Observable state at the beginning of time t. Note that $\mathcal{H}_t$ corresponds to all the information the decision maker has at time t about the state of the infection in the target individual. $\mathcal{S}_t = (\mathcal{H}_{t-1}, (x_\tau)_{\tau \leq t-1})$.

An individual who is infected will be contagious only when the viral load surpasses $L^I = 10^6$ cp/ml and remains active (not isolated) until positive test results emerge or at symptoms onset. This means that an infectious day will occur if and only if the following three events happen at day t:

- $I_t = \{V_t \geq L^I\}$: The individual is infecting.

- $N_t = \{(R_k)_{k \leq t} \in \{-1, 0\}^t\}$: All test results up to day t have been negative.

- Z_t: No symptoms at day t.

Thus, the total number of days where the agent is infecting is equal to:

$$\sum_{t=0}^{T} \mathbb{1}\{I_t \cap N_t \cap Z_t\}.$$

The decision maker designing the test schedule does not know the value of $\mathbb{1}\{I_t \cap N_t \cap Z_t\}$ and can only infer the distribution of the event $I_t \cap N_t \cap Z_t$ based on prior knowledge of the distribution of the viral load for an infected individual as well as the information obtained through the testing policy, which allows to update the belief on the viral load distribution each time a test result is observed.

Define $\mathcal{J}_T = \mathbb{1}\{I_T \cap N_T \cap Z_T\}$ and $\mathcal{J}_t = \mathbb{1}\{I_t \cap N_t \cap Z_t\} + \mathcal{J}_{t+1}$ recursively. Therefore, the decision maker will try at the beginning of each day t to minimize the following quantity:

$$\mathbb{E}[\mathcal{J}_t \mid \mathcal{S}_t] = \mathbb{E}[\mathbb{1}\{I_t \cap N_t \cap Z_t\} \mid \mathcal{S}_t] + \mathbb{E}[\mathcal{J}_{t+1} \mid \mathcal{S}_t]. \tag{3}$$

Recall that $\mathcal{H}_t = \sigma(R_k^A, R_k^P \mid k \leq t)$, which means that N_{t-1} is $\mathcal{H}_{t-1}$-measurable, and since $N_t = N_{t-1} \cap \{R_t \in \{-1, 0\}\}$, we can rewrite the first term of Equation (3) as

$$\mathbb{E}[\mathbb{1}\{I_t \cap N_t \cap Z_t\} \mid \mathcal{S}_t] = \mathbb{1}\{N_{t-1}, Z_t\}\mathbb{E}[\mathbb{1}\{I_t \cap \{R_t \in \{-1, 0\}\} \cap Z_t\} \mid \mathcal{S}_t] = \mathbb{P}(I_t, R_t \in \{-1, 0\} \mid \mathcal{S}_t),$$

because if a positive test is observed at some point in the past, the individual is taken to quarantine and the risk is over, then $\mathbb{1}\{N_{t-1}\}$ must be equal to one if the decision maker is making a decision at time t. The same happens if the individual presents symptoms on day t. Given that the distribution of R_t is determined by x_t^A and x_t^P, we have

$$\mathbb{P}(I_t, R_t \in \{-1, 0\}, Z_t \mid \mathcal{S}_t) = \begin{cases} \mathbb{P}(V_t \geq L^I \mid \mathcal{S}_t) & x_t^P = x_t^A = 0 \\ \mathbb{P}(V_t \geq L^I, V_t < L^A \mid \mathcal{S}_t) = 0 & x_t^A = 1 \text{ and } x_t^P = 0 \\ \mathbb{P}(V_t \geq L^I, V_{t-1} < L^P \mid \mathcal{S}_t) & x_t^A = 0 \text{ and } x_t^P = 1 \\ \mathbb{P}(V_t \geq L^I, V_{t-1} < L^P, V_t < L^A \mid \mathcal{S}_t\} = 0 & x_t^A = x_t^P = 1 \end{cases} \tag{4}$$

The second and fourth cases are equal to zero since a negative LFA test immediately discards the event that the agent may be infecting. Let us look at the first case in more detail. We have

$$\mathbb{P}(V_t \geq L^I \mid \mathcal{H}_{t-1}, x_1, \ldots, x_{t-1}) = \mathbb{P}(V_t \geq L^I \mid V_{d^A} < L^A \ \forall \in \mathcal{D}^A, \ V_{d^P-1} < L^P \ \forall d^P \in \mathcal{D}^P) \tag{5}$$

The third term in Equation (4) can be written similarly.

Using the probability distributions for the times of LOD and the times for peak viral load and end of contagious period described in Section A, we determine the probability distribution for V_t for each t. Thus, we can use Monte Carlo simulations to compute the value of (5) using the identity:

$$\begin{aligned}&\mathbb{P}(V_t \geq L^I \mid V_{d^A} < L^A \ \forall \in \mathcal{D}^A,\ V_{d^P-1} < L^P \ \forall d^P \in \mathcal{D}^P) \\ &= \frac{\mathbb{P}(V_t \geq L^I, V_{d^A} < L^A \ \forall \in \mathcal{D}^A,\ V_{d^P-1} < L^P \ \forall d^P \in \mathcal{D}^P)}{\mathbb{P}(V_{d^A} < L^A \ \forall \in \mathcal{D}^A,\ V_{d^P-1} < L^P \ \forall d^P \in \mathcal{D}^P)}.\end{aligned} \tag{6}$$

To conclude, we recall that the expected number of infected days is given by

$$\mathbb{E}\left[\sum_{t=0}^{T} \mathbb{1}\{I_t \cap N_t \cap Z_t\}\right] = \sum_{t=0}^{T} \mathbb{P}(I_t, N_t, Z_t).$$

By total probability, we can condition each of the probabilities $\mathbb{P}(I_t, N_t, Z_t)$ by the state up to time t, which indicates the probability of being in such a state if we follow a certain policy. Each of these terms is of the form $\mathbb{P}(I_t, N_t, Z_t \mid \mathcal{S}_t)\mathbb{P}(\mathcal{S}_t)$; thus, in Equation (6), the expectation can be written as

$$\mathbb{E}\left[\sum_{t=0}^{T} \mathbb{1}\{I_t \cap N_t \cap Z_t\}\right] = \sum_{t=0}^{T} \mathbb{P}(V_t \geq L^I, V_{d^A} < L^A \ \forall \in \mathcal{D}^A,\ V_{d^P-1} < L^P \ \forall d^P \in \mathcal{D}^P)$$

where each of the terms in the summation can be computed using Monte Carlo simulations.

D Additional results

D.1 Analysis with other types of index case detection

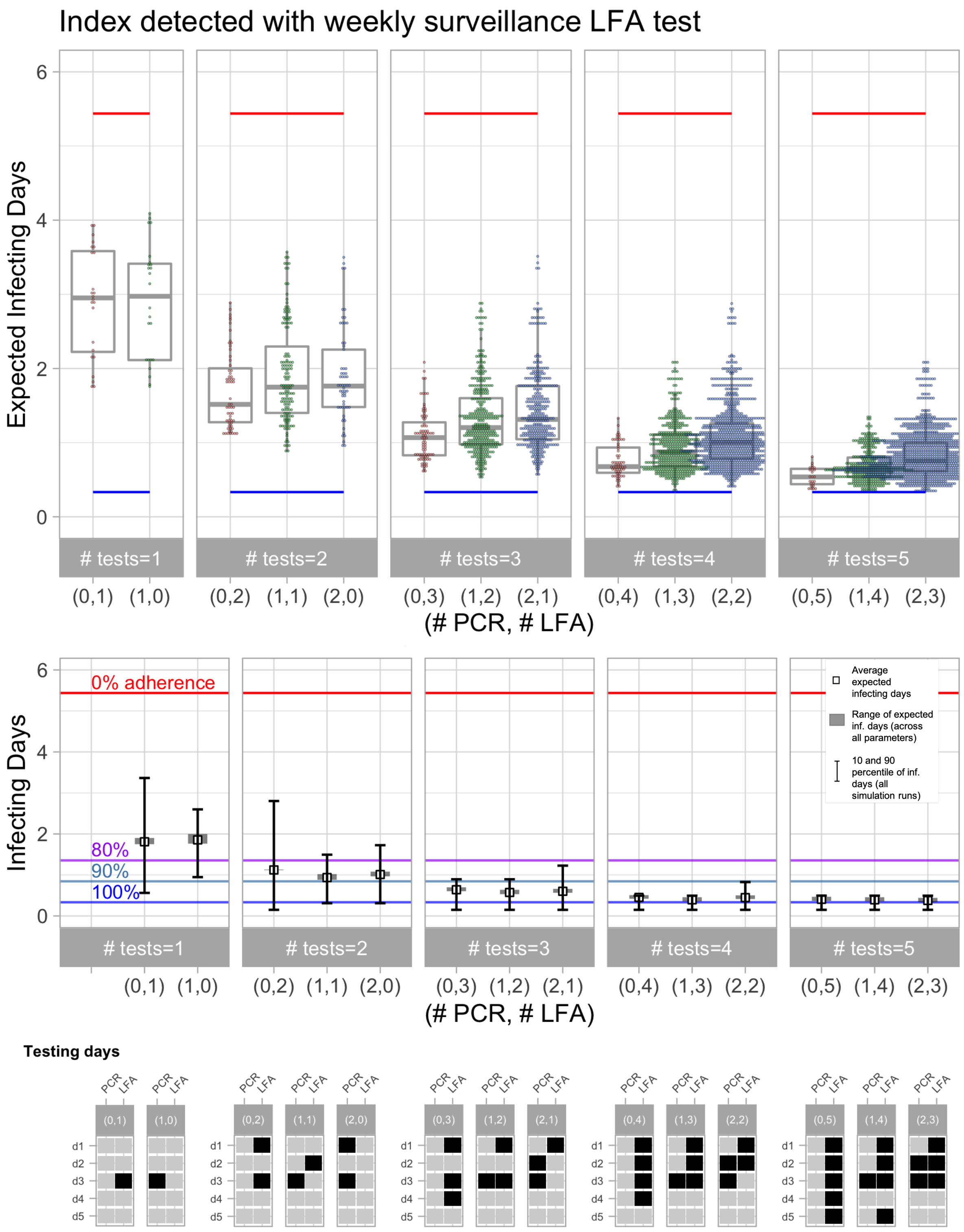

Figure D.1.1: Evaluation of testing strategies for a traced contact exposed to an index case detected with weekly surveillance LFA tests.

D.2 Results with different scenarios of LFA test sensitivity

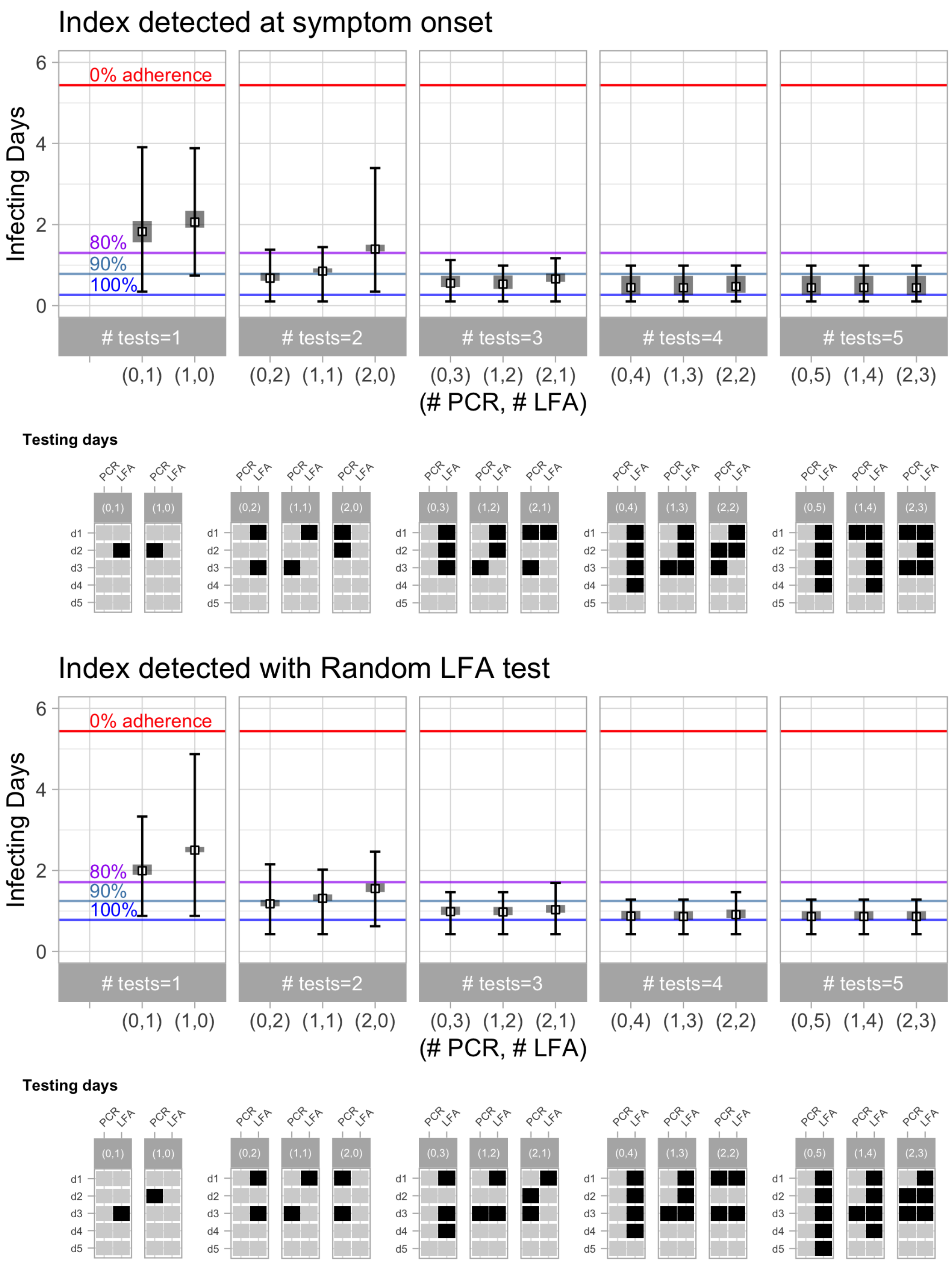

Figure D.2.1: Robust testing policies for the *LFA High* test sensitivity scenario. For the test schedule with (PCR=0,LFA=5) in the top panel, the last LFA test is on day 6 (not shown in the figure).

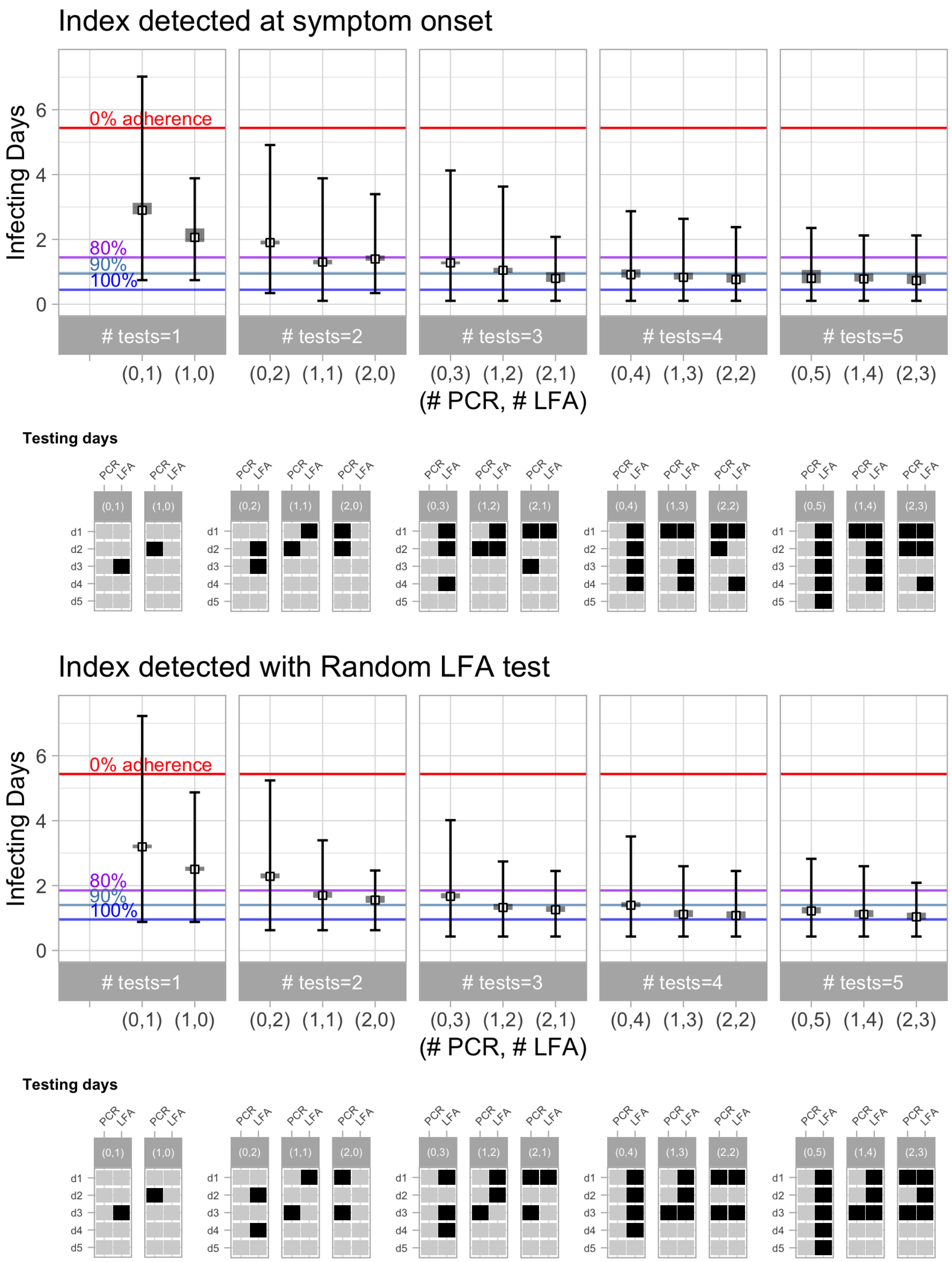

Figure D.2.2: Robust testing policies for the *LFA Low* test sensitivity scenario. For the test schedule (PCR=1,LFA=4) in the bottom panel, the last LFA is taken on day 6 (not shown in the figure).

D.3 Results with alternative models of viral load trajectories

Figure D.3.1 compares the infectiousness and test sensitivity of the grouped estimates of viral load trajectories reported in [1], with those used in our main analysis (based on [2]). Specifically, we simulated viral load paths using the group estimates reported in Figure S5 in the Supplementary material of [1], summarized in Table D.3.1 (each viral load path was generated by simulating each parameter from a Normal distribution with the indicated mean and standard deviation). These viral load trajectories sug-

Parameter	Mean	Std. Dev.
Increasing slope	2	0.39
Days to peak load	4.3	0.92
Peak viral load	8.1	0.7
Decreasing slope	-0.17	0.02

Table D.3.1: Parameters to simulate viral load trajectories based on the results reported in [1]

gest a more extended period of infectiousness, but also earlier detection with PCR and LFA tests and symptoms onset. To study whether this affected the main conclusions of our analysis, we repeated all the simulations using the viral model of [1].

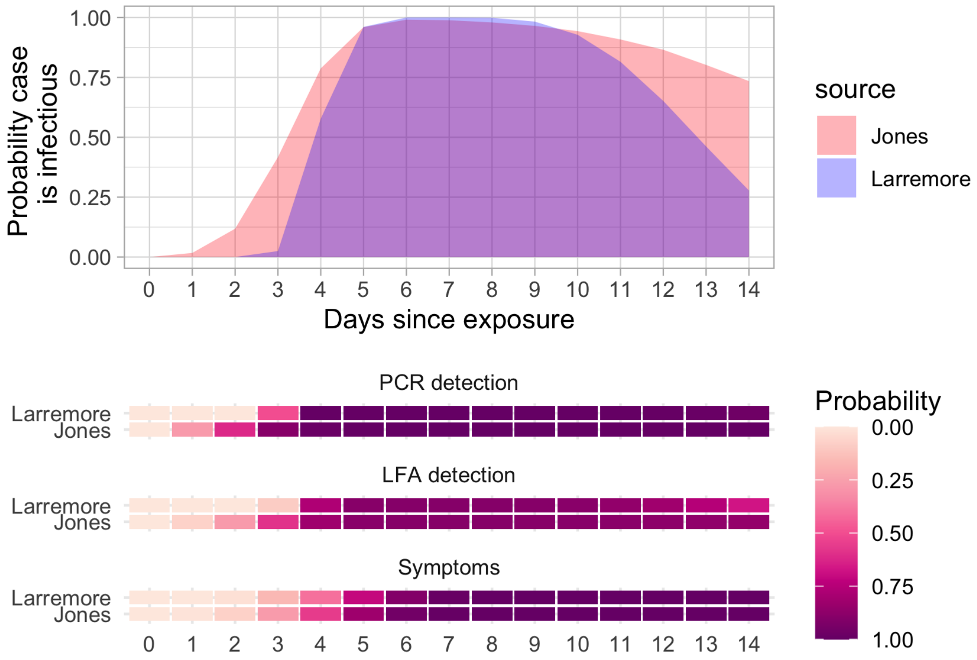

Figure D.3.1: Comparison of infectiousness and test sensitivity dynamics between the viral load models of [2] and [1]

.

Figures D.3.2 and D.3.3 show the robust testing policies obtained through the simulations using these alternative viral load paths, considering two scenarios of LFA test sensitivity (*LFA Med* and *LFA Med-Low*).

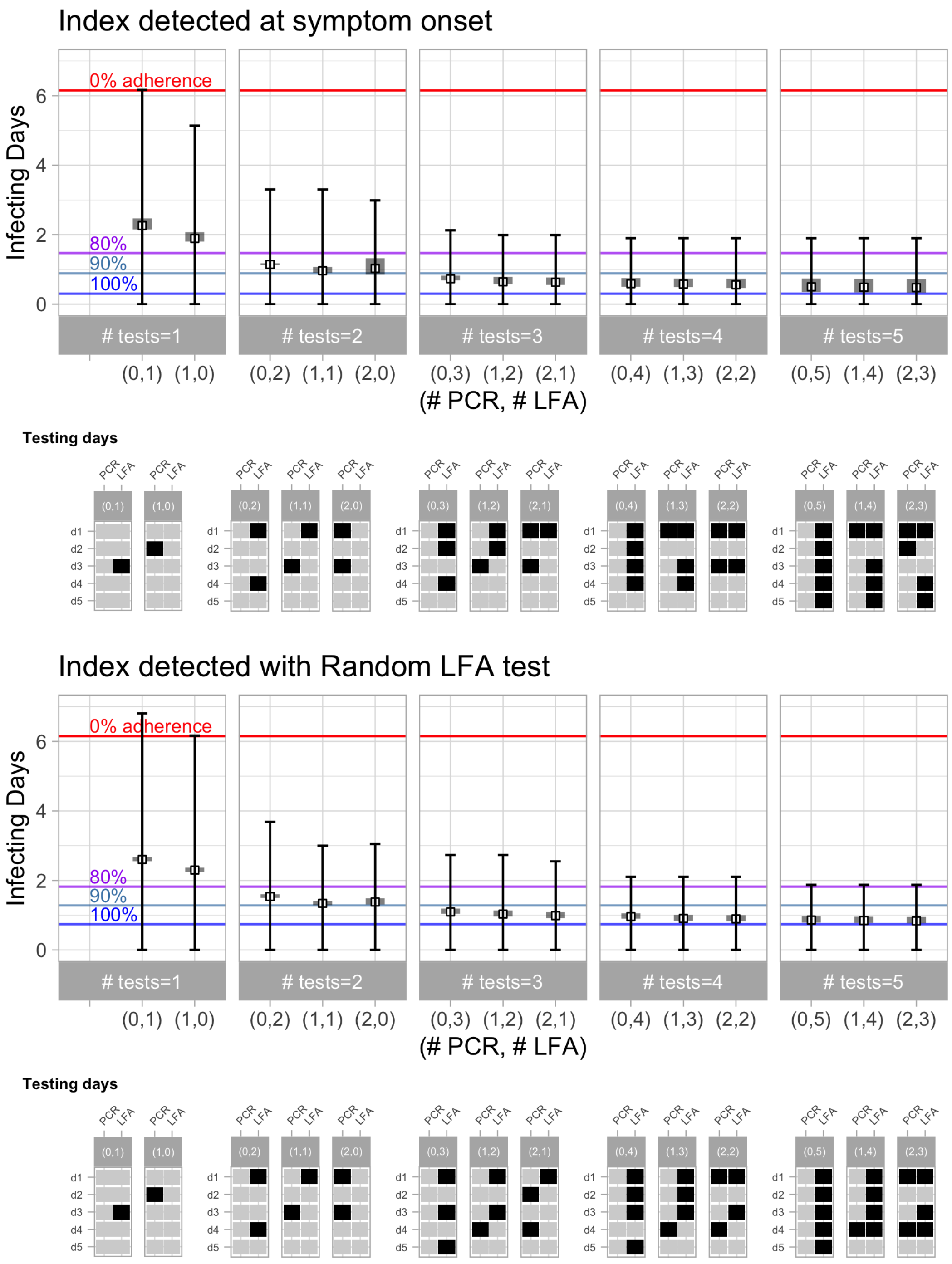

Figure D.3.2: Robust testing policies for the *LFA Med* test sensitivity scenario using viral load trajectories from [1].

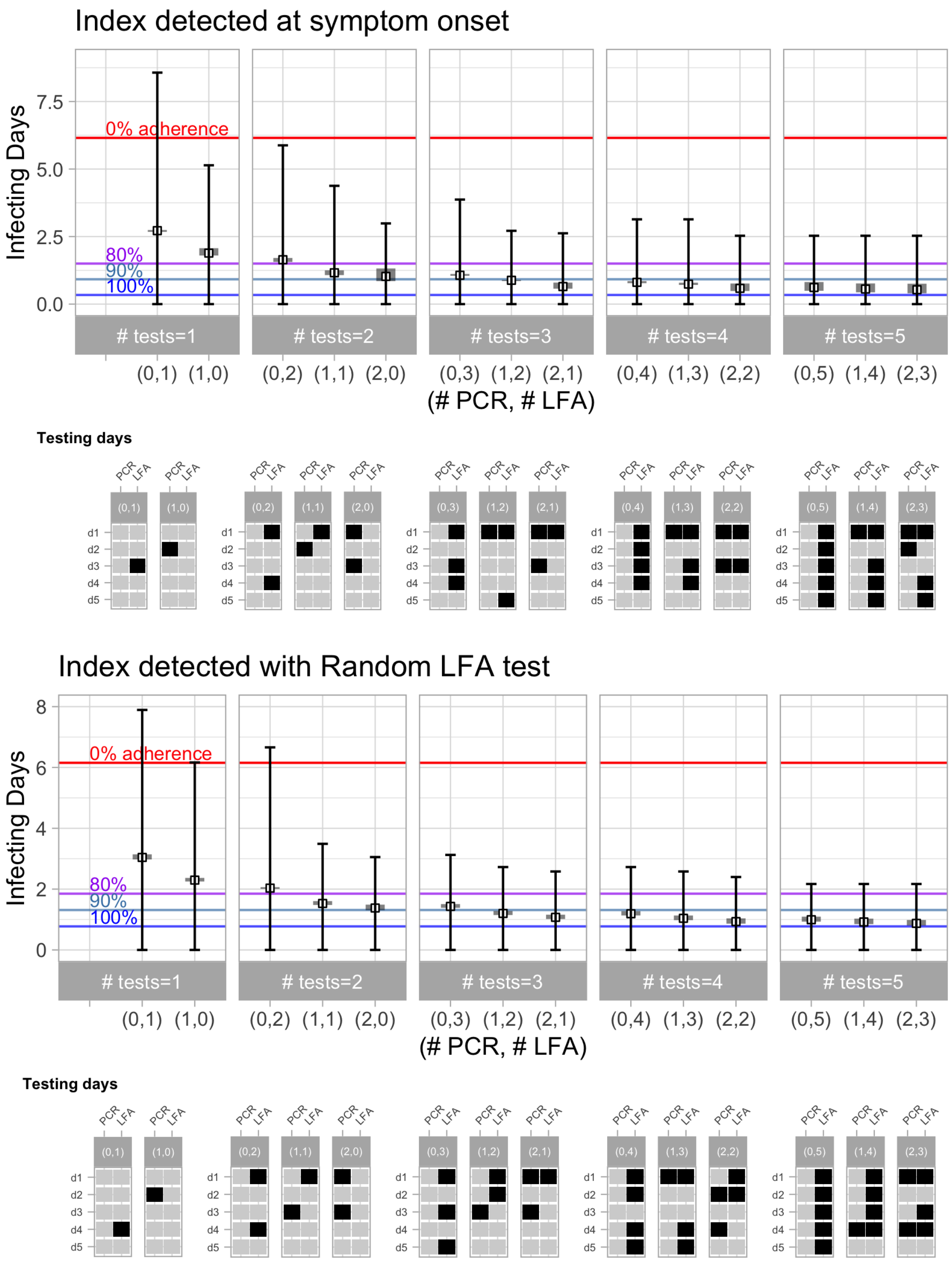

Figure D.3.3: Robust testing policies for the *LFA Med-Low* test sensitivity scenario using viral load trajectories from [1].

D.4 Detailed results of robust policies

In what follows, we present the expected infecting days and the false negative rates at the times when tests are performed, for all values of the infectivity parameter β and all combinations of tests considered. For Tables D.4.1, D.4.2, and D.4.3, we considered the LFA *Med* test sensitivity scenario.

# LFA	# PCR	LFA schedule	PCR schedule	Exp.infecting days	β	FNR (LFA)	FNR (PCR)
1	0	[2]		2.07	0.01	[0.14]	
1	0	[2]		2.13	0.1	[0.14]	
1	0	[2]		2.42	0.5	[0.12]	
1	0	[2]		2.82	1	[0.10]	
2	0	[1, 3]		1.08	0.01	[0.31, 0.11]	
2	0	[1, 3]		1.11	0.1	[0.29, 0.11]	
2	0	[1, 3]		1.34	0.5	[0.20, 0.10]	
2	0	[1, 3]		1.68	1	[0.12, 0.10]	
3	0	[1, 2, 3]		0.77	0.01	[0.31, 0.16, 0.12]	
3	0	[1, 2, 3]		0.83	0.1	[0.29, 0.16, 0.12]	
3	0	[1, 2, 3]		1.13	0.5	[0.20, 0.13, 0.11]	
3	0	[1, 2, 3]		1.55	1	[0.12, 0.11, 0.11]	
4	0	[1, 2, 3, 4]		0.71	0.01	[0.31, 0.16, 0.12, 0.11]	
4	0	[1, 2, 3, 4]		0.78	0.1	[0.29, 0.16, 0.12, 0.11]	
4	0	[1, 2, 3, 4]		1.11	0.5	[0.20, 0.13, 0.11, 0.10]	
4	0	[1, 2, 3, 4]		1.55	1	[0.12, 0.11, 0.10, 0.11]	
5	0	[1, 2, 3, 4, 5]		0.71	0.01	[0.31, 0.16, 0.12, 0.10, 0.06]	
5	0	[1, 2, 3, 4, 5]		0.77	0.1	[0.29, 0.16, 0.12, 0.10 , 0.06]	
5	0	[1, 2, 3, 4, 5]		1.11	0.5	[0.20, 0.13, 0.11, 0.09, 0.04]	
5	0	[1, 2, 3, 4, 5]		1.54	1	[0.12, 0.11, 0.11, 0.09, 0.02]	
0	1		[2]	1.37	0.01		[0.02]
0	1		[2]	1.44	0.1		[0.02]
0	1		[2]	1.83	0.5		[0.01]
0	1		[2]	2.33	1		[0.00]
1	1	[1]	[3]	0.87	0.01	[0.31]	[0.00]
1	1	[1]	[3]	0.92	0.1	[0.29]	[0.00]
1	1	[1]	[3]	1.22	0.5	[0.20]	[0.00]
1	1	[1]	[3]	1.63	1	[0.12]	[0.00]
2	1	[1, 2]	[3]	0.71	0.01	[0.31, 0.16]	[0.01]
2	1	[1, 2]	[3]	0.77	0.1	[0.29, 0.16]	[0.01]
2	1	[1, 2]	[3]	1.11	0.5	[0.20, 0.13]	[0.00]
2	1	[1, 2]	[3]	1.54	1	[0.12, 0.11]	[0.00]
3	1	[1, 2, 3]	[1]	0.7	0.01	[0.31, 0.19, 0.10]	[0.35]
3	1	[1, 2, 3]	[1]	0.75	0.1	[0.29, 0.18, 0.09]	[0.32]
3	1	[1, 2, 3]	[1]	1.04	0.5	[0.20, 0.13, 0.07]	[0.17]
3	1	[1, 2, 3]	[1]	1.45	1	[0.12, 0.08, 0.04]	[0.04]
4	1	[1, 2, 3, 4]	[1]	0.64	0.01	[0.31, 0.19 , 0.10, 0.05]	[0.35]
4	1	[1, 2, 3, 4]	[1]	0.7	0.1	[0.29, 0.18, 0.09, 0.05]	[0.32]
4	1	[1, 2, 3, 4]	[1]	1.02	0.5	[0.20, 0.14, 0.07, 0.03]	[0.17]
4	1	[1, 2, 3, 4]	[1]	1.45	1	[0.12, 0.08, 0.07, 0.01]	[0.04]
5	1	[1, 2, 3, 4, 5]	[1]	0.64	0.01	[0.31, 0.18, 0.10, 0.05, 0.02]	[0.35]
5	1	[1, 2, 3, 4, 5]	[1]	0.7	0.1	[0.29, 0.18, 0.09, 0.05, 0.02]	[0.32]
5	1	[1, 2, 3, 4, 5]	[1]	1.02	0.5	[0.20, 0.14, 0.07, 0.03, 0.04]	[0.17]
5	1	[1, 2, 3, 4, 5]	[1]	1.45	1	[0.12, 0.08, 0.04, 0.01, 0.00]	[0.04]
0	2		[1, 2]	0.77	0.01		[0.28, 0.05]
0	2		[1, 2]	0.82	0.1		[0.25, 0.04]
0	2		[1, 2]	1.07	0.5		[0.11, 0.03]
0	2		[1, 2]	1.45	1		[0.01, 0.02]
1	2	[1]	[1, 3]	0.72	0.01	[0.31]	[0.35, 0.00]
1	2	[1]	[1, 3]	0.77	0.1	[0.29]	[0.32, 0.00]
1	2	[1]	[1, 3]	1.05	0.5	[0.20]	[0.17, 0.00]
1	2	[1]	[1, 3]	1.45	1	[0.12]	[0.04, 0.00]
2	2	[1, 2]	[1, 3]	0.64	0.01	[0.31, 0.19]	[0.35 0.01]
2	2	[1, 2]	[1, 3]	0.7	0.1	[0.29, 0.18]	[0.31, 0.01]
2	2	[1, 2]	[1, 3]	1.02	0.5	[0.20, 0.143]	[0.17, 0.00]
2	2	[1, 2]	[1, 3]	1.45	1	[0.12, 0.08]	[0.04, 0.00]
3	2	[1, 2, 4]	[1, 2]	0.64	0.01	[0.31, 0.18, 0.06]	[0.35, 0.18]
3	2	[1, 2, 4]	[1, 2]	0.7	0.1	[0.29, 0.18, 0.05]	[0.32, 0.16]
3	2	[1, 2, 4]	[1, 2]	1.02	0.5	[0.20, 0.14, 0.03]	[0.17, 0.11]
3	2	[1, 2, 4]	[1, 2]	1.45	1	[0.12, 0.08, 0.01]	[0.04, 0.06]
4	2	[1, 2, 3, 4]	[1, 2]	0.63	0.01	[0.31, 0.18, 0.12, 0.08]	[0.35, 0.17]
4	2	[1, 2, 3, 4]	[1, 2]	0.69	0.1	[0.29, 0.18, 0.11, 0.05]	[0.32, 0.16]
4	2	[1, 2, 3, 4]	[1, 2]	1.01	0.5	[0.20, 0.14, 0.07, 0.03]	[0.17, 0.11]
4	2	[1, 2, 3, 4]	[1, 2]	1.45	1	[0.12, 0.08, 0.02, 0.01]	[0.04, 0.06]
5	2	[1, 2, 3, 4, 6]	[1, 2]	0.62	0.01	[0.31 0.19, 0.12, 0.06, 0.02]	[0.35, 0.17]
5	2	[1, 2, 3, 4, 6]	[1, 2]	0.69	0.1	[0.29, 0.18, 0.11, 0.06, 0.02]	[0.32, 0.16]
5	2	[1, 2, 3, 4, 6]	[1, 2]	1.01	0.5	[0.20, 0.13, 0.07, 0.03, 0.01]	[0.17, 0.14]
5	2	[1, 2, 3, 4, 6]	[1, 2]	1.45	1	[0.12, 0.08, 0.01, 0.01, 0.00]	[0.04, 0.06]

Table D.4.1: Index detected at symptom onset

# LFA	# PCR	LFA schedule	PCR schedule	Exp.infecting days	β	FNR (LFA)	FNR (PCR)
1	0	[3]		2.93	0.01	[0.11]	
1	0	[3]		2.96	0.1	[0.11]	
1	0	[3]		3.07	0.5	[0.11]	
1	0	[3]		3.21	1	[0.11]	
2	0	[1, 3]		1.62	0.01	[0.33, 0.11]	
2	0	[1, 3]		1.64	0.1	[0.33, 0.11]	
2	0	[1, 3]		1.73	0.5	[0.31, 0.11]	
2	0	[1, 3]		1.84	1	[0.29, 0.11]	
3	0	[1, 3, 4]		1.45	0.01	[0.33, 0.11, 0.11]	
3	0	[1, 3, 4]		1.47	0.1	[0.33, 0.11, 0.11]	
3	0	[1, 3, 4]		1.57	0.5	[0.31, 0.11, 0.12]	
3	0	[1, 3, 4]		1.7	1	[0.29, 0.11, 0.12]	
4	0	[1, 2, 3, 4]		1.25	0.01	[0.33, 0.17, 0.13, 0.11]	
4	0	[1, 2, 3, 4]		1.28	0.1	[0.33, 0.17, 0.13, 0.12]	
4	0	[1, 2, 3, 4]		1.38	0.5	[0.31, 0.16, 0.13, 0.12]	
4	0	[1, 2, 3, 4]		1.53	1	[0.29, 0.16, 0.13, 0.12]	
5	0	[1, 2, 3, 4, 5]		1.25	0.01	[0.33, 0.17, 0.13, 0.12, 0.06]	
5	0	[1, 2, 3, 4, 5]		1.27	0.1	[0.33, 0.17, 0.13, 0.12, 0.06]	
5	0	[1, 2, 3, 4, 5]		1.38	0.5	[0.31, 0.16, 0.13, 0.12, 0.07]	
5	0	[1, 2, 3, 4, 5]		1.52	1	[0.29, 0.16, 0.13, 0.12, 0.09]	
0	1		[2]	1.86	0.01		[0.03]
0	1		[2]	1.89	0.1		[0.03]
0	1		[2]	2	0.5		[0.02]
0	1		[2]	2.14	1		[0.02]
1	1	[1]	[3]	1.4	0.01	[0.33]	[0.00]
1	1	[1]	[3]	1.43	0.1	[0.33]	[0.00]
1	1	[1]	[3]	1.53	0.5	[0.31]	[0.00]
1	1	[1]	[3]	1.66	1	[0.29]	[0.00]
2	1	[1, 2]	[3]	1.25	0.01	[0.33, 0.17]	[0.01]
2	1	[1, 2]	[3]	1.27	0.1	[0.33, 0.17]	[0.01]
2	1	[1, 2]	[3]	1.38	0.5	[0.31, 0.16]	[0.01]
2	1	[1, 2]	[3]	1.52	1	[0.29, 0.16]	[0.01]
3	1	[1, 2, 3]	[3]	1.25	0.01	[0.33, 0.17, 0.13]	[0.03]
3	1	[1, 2, 3]	[3]	1.27	0.1	[0.33, 0.17, 0.13]	[0.03]
3	1	[1, 2, 3]	[3]	1.38	0.5	[0.31, 0.16, 0.13]	[0.02]
3	1	[1, 2, 3]	[3]	1.52	1	[0.29, 0.16, 0.13]	[0.02]
4	1	[1, 2, 3, 5]	[3]	1.24	0.01	[0.33, 0.17, 0.13, 0.03]	[0.03]
4	1	[1, 2, 3, 5]	[3]	1.27	0.1	[0.33, 0.17, 0.13, 0.03]	[0.03]
4	1	[1, 2, 3, 5]	[3]	1.37	0.5	[0.31, 0.16, 0.13, 0.03]	[0.02]
4	1	[1, 2, 3, 5]	[3]	1.52	1	[0.29, 0.16, 0.13, 0.02]	[0.02]
5	1	[1, 2, 3, 4, 5]	[2]	1.22	0.01	[0.33, 0.17, 0.12, 0.06, 0.03]	[0.13]
5	1	[1, 2, 3, 4, 5]	[2]	1.25	0.1	[0.33, 0.17, 0.11, 0.05, 0.03]	[0.13]
5	1	[1, 2, 3, 4, 5]	[2]	1.36	0.5	[0.31, 0.16, 0.11, 0.05, 0.03]	[0.12]
5	1	[1, 2, 3, 4, 5]	[2]	1.5	1	[0.29, 0.16, 0.10, 0.05, 0.02]	[0.10]
0	2		[1, 3]	1.35	0.01		[0.31, 0.00]
0	2		[1, 3]	1.37	0.1		[0.30, 0.00]
0	2		[1, 3]	1.46	0.5		[0.28, 0.00]
0	2		[1, 3]	1.58	1		[0.24, 0.00]
1	2	[1]	[2, 3]	1.23	0.01	[0.33]	[0.04, 0.02]
1	2	[1]	[2, 3]	1.25	0.1	[0.33]	[0.04, 0.02]
1	2	[1]	[2, 3]	1.36	0.5	[0.31]	[0.03, 0.02]
1	2	[1]	[2, 3]	1.51	1	[0.29]	[0.03, 0.02]
2	2	[1, 2]	[2, 3]	1.23	0.01	[0.33, 0.17]	[0.13, 0.03]
2	2	[1, 2]	[2, 3]	1.25	0.1	[0.33, 0.17]	[0.13, 0.03]
2	2	[1, 2]	[2, 3]	1.36	0.5	[0.31, 0.16]	[0.12, 0.03]
2	2	[1, 2]	[2, 3]	1.5	1	[0.29, 0.16]	[0.10, 0.03]
3	2	[1, 2, 3]	[1, 3]	1.18	0.01	[0.33, 0.16, 0.09]	[0.37, 0.03]
3	2	[1, 2, 3]	[1, 3]	1.21	0.1	[0.33, 0.16, 0.09]	[0.36, 0.03]
3	2	[1, 2, 3]	[1, 3]	1.31	0.5	[0.31, 0.15, 0.08]	[0.33 0.03]
3	2	[1, 2, 3]	[1, 3]	1.45	1	[0.29, 0.14, 0.08]	[0.30, 0.02]
4	2	[1, 2, 3, 5]	[1, 3]	1.18	0.01	[0.33, 0.16, 0.09, 0.03]	[0.37, 0.03]
4	2	[1, 2, 3, 5]	[1, 3]	1.2	0.1	[0.33, 0.16, 0.09, 0.03]	[0.36, 0.03]
4	2	[1, 2, 3, 5]	[1, 3]	1.31	0.5	[0.31, 0.15, 0.08, 0.03]	[0.33, 0.03]
4	2	[1, 2, 3, 5]	[1, 3]	1.45	1	[0.29, 0.14, 0.07, 0.02]	[0.30, 0.02]
5	2	[1, 2, 3, 4, 5]	[1, 2]	1.17	0.01	[0.33, 0.16, 0.12, 0.06, 0.03]	[0.37, 0.16]
5	2	[1, 2, 3, 4, 5]	[1, 2]	1.19	0.1	[0.33, 0.16, 0.12, 0.07, 0.03]	[0.36, 0.17]
5	2	[1, 2, 3, 4, 5]	[1, 2]	1.3	0.5	[0.31, 0.15, 0.11, 0.05, 0.02]	[0.33, 0.15]
5	2	[1, 2, 3, 4, 5]	[1, 2]	1.44	1	[0.29, 0.14, 0.10, 0.04, 0.03]	[0.30, 0.16]

Table D.4.2: Index detected with random LFA test

# LFA	# PCR	LFA schedule	PCR schedule	Exp.infecting days	β	FNR (LFA)	FNR (PCR)
1	0	[3]		2.34	0.01	[0.11]	
1	0	[3]		2.35	0.1	[0.11]	
1	0	[3]		2.43	0.5	[0.11]	
1	0	[3]		2.56	1	[0.11]	
2	0	[1, 3]		1.11	0.01	[0.42, 0.11]	
2	0	[1, 3]		1.11	0.1	[0.41, 0.11]	
2	0	[1, 3]		1.15	0.5	[0.39, 0.11]	
2	0	[1, 3]		1.22	1	[0.36, 0.11]	
3	0	[1, 3, 4]		0.87	0.01	[0.42, 0.11, 0.11]	
3	0	[1, 3, 4]		0.88	0.1	[0.41, 0.11, 0.11]	
3	0	[1, 3, 4]		0.94	0.5	[0.39, 0.11, 0.11]	
3	0	[1, 3, 4]		1.01	1	[0.36, 0.11, 0.11]	
4	0	[1, 2, 3, 4]		0.63	0.01	[0.42, 0.19, 0.13, 0.11]	
4	0	[1, 2, 3, 4]		0.65	0.1	[0.41, 0.19, 0.13, 0.11]	
4	0	[1, 2, 3, 4]		0.71	0.5	[0.39, 0.18, 0.13, 0.11]	
4	0	[1, 2, 3, 4]		0.80	1	[0.36, 0.17, 0.13, 0.11]	
5	0	[1, 2, 3, 4, 5]		0.63	0.01	[0.42, 0.19, 0.13, 0.11, 0.04]	
5	0	[1, 2, 3, 4, 5]		0.64	0.1	[0.41, 0.19, 0.13, 0.11, 0.07]	
5	0	[1, 2, 3, 4, 5]		0.70	0.5	[0.39, 0.18, 0.13, 0.11, 0.06]	
5	0	[1, 2, 3, 4, 5]		0.79	1	[0.36, 0.17, 0.13, 0.11, 0.06]	
0	1		[1]	1.76	0.01		[0.0]
0	1		[1]	1.78	0.1		[0.0]
0	1		[1]	1.87	0.5		[0.0]
0	1		[1]	2.00	1		[0.0]
1	1	[2]	[3]	1.09	0.01	[0.16]	[0.01]
1	1	[2]	[3]	1.11	0.1	[0.16]	[0.01]
1	1	[2]	[3]	1.20	0.5	[0.16]	[0.01]
1	1	[2]	[3]	1.32	1	[0.15]	[0.01]
2	1	[1, 3]	[3]	0.81	0.01	[0.42, 0.11]	[0.01]
2	1	[1, 3]	[3]	0.82	0.1	[0.41, 0.11]	[0.01]
2	1	[1, 3]	[3]	0.88	0.5	[0.39, 0.11]	[0.01]
2	1	[1, 3]	[3]	0.96	1	[0.36, 0.11]	[0.01]
3	1	[1, 2, 3]	[3]	0.62	0.01	[0.42, 0.19, 0.13]	[0.04]
3	1	[1, 2, 3]	[3]	0.64	0.1	[0.41, 0.19, 0.13]	[0.04]
3	1	[1, 2, 3]	[3]	0.70	0.5	[0.39, 0.18, 0.13]	[0.03]
3	1	[1, 2, 3]	[3]	0.79	1	[0.36, 0.17, 0.13]	[0.03]
4	1	[1, 2, 3, 5]	[3]	0.62	0.01	[0.42, 0.19, 0.13, 0.04]	[0.04]
4	1	[1, 2, 3, 5]	[3]	0.63	0.1	[0.41, 0.19, 0.13, 0.04]	[0.04]
4	1	[1, 2, 3, 5]	[3]	0.70	0.5	[0.39, 0.18, 0.13, 0.04]	[0.03]
4	1	[1, 2, 3, 5]	[3]	0.78	1	[0.36, 0.17, 0.13, 0.04]	[0.03]
5	1	[1, 2, 3, 4, 5]	[2]	0.60	0.01	[0.42, 0.19, 0.16, 0.08, 0.04]	[0.18]
5	1	[1, 2, 3, 4, 5]	[2]	0.61	0.1	[0.41, 0.19, 0.16, 0.09, 0.05]	[0.17]
5	1	[1, 2, 3, 4, 5]	[2]	0.68	0.5	[0.39, 0.18, 0.14, 0.07, 0.04]	[0.16]
5	1	[1, 2, 3, 4, 5]	[2]	0.76	1	[0.36, 0.17, 0.13, 0.06, 0.03]	[0.15]
0	2		[1, 3]	0.79	0.01		[0.43, 0.00]
0	2		[1, 3]	0.80	0.1		[0.42, 0.00]
0	2		[1, 3]	0.85	0.5		[0.39, 0.00]
0	2		[1, 3]	0.91	1		[0.35, 0.00]
1	2	[1]	[2, 3]	0.61	0.01	[0.42]	[0.05, 0.03]
1	2	[1]	[2, 3]	0.62	0.1	[0.41]	[0.05, 0.03]
1	2	[1]	[2, 3]	0.69	0.5	[0.39]	[0.05, 0.03]
1	2	[1]	[2, 3]	0.77	1	[0.36]	[0.04, 0.03]
2	2	[1, 2]	[2, 3]	0.60	0.01	[0.42, 0.19]	[0.18, 0.04]
2	2	[1, 2]	[2, 3]	0.62	0.1	[0.41, 0.19]	[0.17, 0.04]
2	2	[1, 2]	[2, 3]	0.68	0.5	[0.39, 0.18]	[0.16, 0.04]
2	2	[1, 2]	[2, 3]	0.77	1	[0.36, 0.17]	[0.15, 0.03]
3	2	[1, 2, 3]	[2, 3]	0.60	0.01	[0.42, 0.19, 0.16]	[0.18, 0.09]
3	2	[1, 2, 3]	[2, 3]	0.61	0.1	[0.41, 0.19, 0.16]	[0.17, 0.09]
3	2	[1, 2, 3]	[2, 3]	0.68	0.5	[0.39, 0.18, 0.15]	[0.16, 0.11]
3	2	[1, 2, 3]	[2, 3]	0.77	1	[0.36, 0.17, 0.13]	[0.15, 0.08]
4	2	[1, 2, 3, 6]	[2, 3]	0.60	0.01	[0.42, 0.19, 0.15, 0.04]	[0.18, 0.13]
4	2	[1, 2, 3, 6]	[2, 3]	0.61	0.1	[0.41, 0.19, 0.15, 0.04]	[0.18, 0.09]
4	2	[1, 2, 3, 6]	[2, 3]	0.67	0.5	[0.39, 0.18, 0.15, 0.03]	[0.16, 0.09]
4	2	[1, 2, 3, 6]	[2, 3]	0.76	1	[0.36, 0.17, 0.14, 0.03]	[0.15, 0.08]
5	2	[1, 2, 3, 4, 5]	[1, 2]	0.55	0.01	[0.42, 0.21, 0.16, 0.07, 0.04]	[0.50, 0.22]
5	2	[1, 2, 3, 4, 5]	[1, 2]	0.56	0.1	[0.41, 0.2, 0.15, 0.08, 0.05]	[0.49, 0.21]
5	2	[1, 2, 3, 4, 5]	[1, 2]	0.63	0.5	[0.39, 0.2, 0.15, 0.09, 0.04]	[0.46, 0.20]
5	2	[1, 2, 3, 4, 5]	[1, 2]	0.71	1	[0.36, 0.19, 0.14, 0.06, 0.03]	[0.42, 0.19]

Table D.4.3: Index case detected with weekly surveillance LFA test

References

[1] T. C. Jones, G. Biele, B. Mühlemann, T. Veith, J. Schneider, J. Beheim-Schwarzbach, T. Bleicker, J. Tesch, M. L. Schmidt, L. E. Sander, et al. Estimating infectiousness throughout sars-cov-2 infection course. *Science*, 2021.

[2] D. B. Larremore, B. Wilder, E. Lester, S. Shehata, J. M. Burke, J. A. Hay, M. Tambe, M. J. Mina, and R. Parker. Test sensitivity is secondary to frequency and turnaround time for covid-19 screening. *Science advances*, 7(1):eabd5393, 2021.
